# Role of cell rearrangement and related signaling pathways in the dynamic process of tip cell selection

**DOI:** 10.1186/s12964-023-01364-1

**Published:** 2024-01-09

**Authors:** Yaru Guo, Shihan Zhang, Dandan Wang, Boon Chin Heng, Xuliang Deng

**Affiliations:** 1 Department of Geriatric Dentistry, Peking University School and Hospital of Stomatology, Beijing, 100081 P. R. China; 2 Department of Pediatric Dentistry, Peking University School and Hospital of Stomatology, Beijing, 100081 China; 3 Central Laboratory, Peking University School and Hospital of Stomatology, Beijing, 100081 China; 4 NMPA Key Laboratory for Dental Materials, Department of Dental Materials & Dental Medical Devices Testing Center, Peking University School and Hospital of Stomatology, Beijing, 100081 China; 5 National Engineering Research Center of Oral Biomaterials and Digital Medical Devices, Beijing, China; 6 Laboratory of Biomedical Materials, Peking University School and Hospital of Stomatology, Beijing, 100081 China

**Keywords:** Tip cell, Cell rearrangement, Angiogenesis, Notch, YAP/TAZ

## Abstract

Angiogenesis is a complex, highly-coordinated and multi-step process of new blood vessel formation from pre-existing blood vessels. When initiated, the sprouting process is spearheaded by the specialized endothelial cells (ECs) known as tip cells, which guide the organization of accompanying stalk cells and determine the function and morphology of the finally-formed blood vessels. Recent studies indicate that the orchestration and coordination of angiogenesis involve dynamic tip cell selection, which is the competitive selection of cells to lead the angiogenic sprouts. Therefore, this review attempt to summarize the underlying mechanisms involved in tip cell specification in a dynamic manner to enable readers to gain a systemic and overall understanding of tip cell formation, involving cooperative interaction of cell rearrangement with Notch and YAP/TAZ signaling. Various mechanical and chemical signaling cues are integrated to ensure the right number of cells at the right place during angiogenesis, thereby precisely orchestrating morphogenic functions that ensure correct patterning of blood vessels.

Video Abstract

**Supplementary Information:**

The online version contains supplementary material available at 10.1186/s12964-023-01364-1.

## Background

**Fig. 1:**
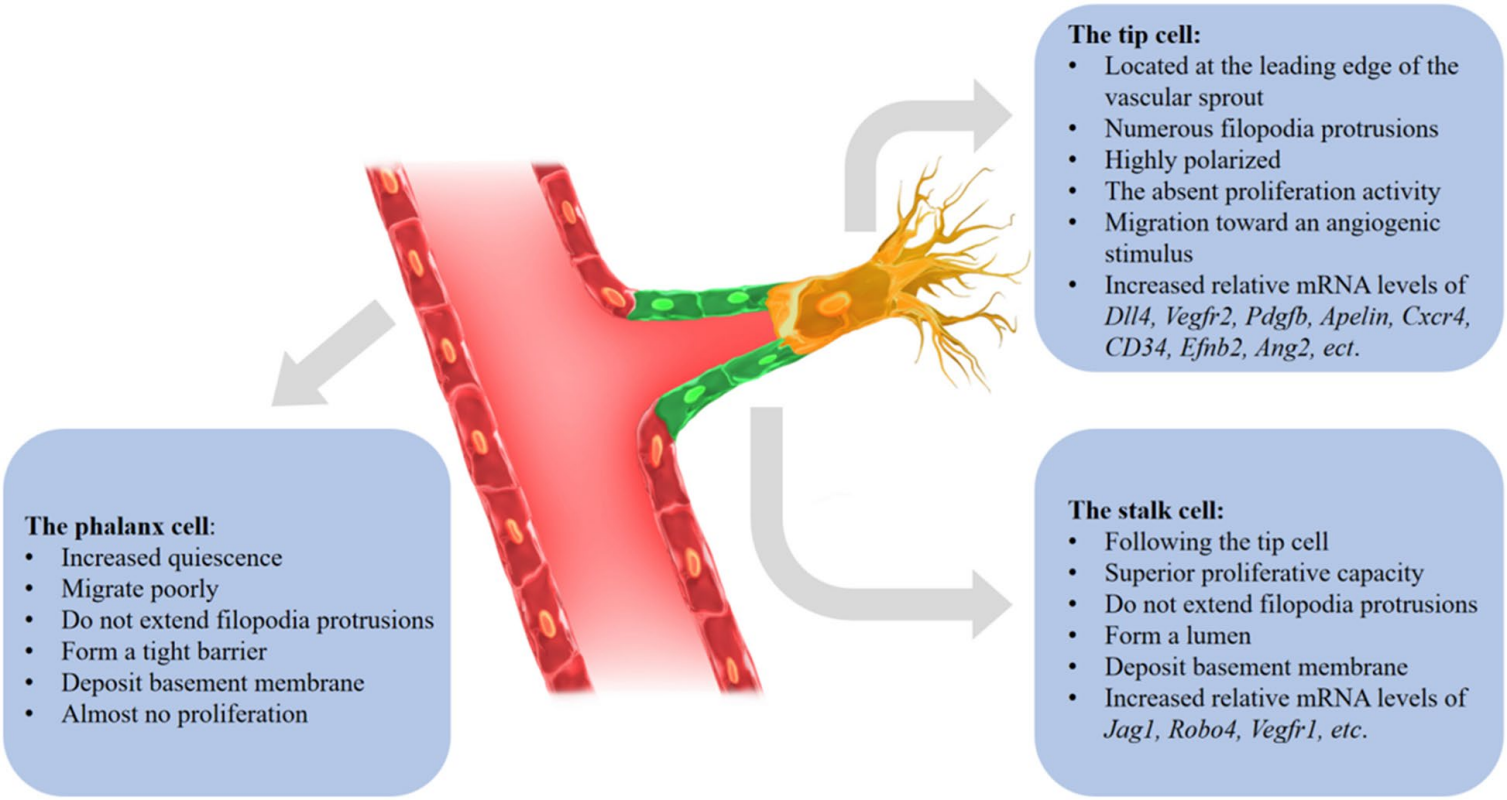
Phenotypic and molecular differences among endothelial tip cells, stalk cells and phalanx cells

Angiogenesis is a highly-coordinated process by which endothelial cells (EC) sprouting from pre-existing blood vessels guides the formation of new vessels. It is essential for many physiological and pathological processes, including embryo development, tumorigenesis, proliferative diabetic retinopathy, atherosclerosis and tissue repair [1]. The importance of angiogenesis has aroused the interests of researchers seeking potential therapeutic targets to promote revascularization in ischaemic tissues or block angiogenesis in cancer, skin, joint or ocular disorders. Hence, over the past few decades, concentrated efforts have been made to investigate the highly-complex angiogenesis process. Briefly, an insufficient supply of nutrients and oxygen prompts hypoxic tissues to secrete various growth factors and chemokines, which stimulates ECs to break out of their stable position within the vessel wall and jointly coordinate sprouting, branching, and new lumenized network formation, until supply meets demand and quiescence can be re-established [2]. Once initiated by growth factor signals within the pathological environment, in particular vascular endothelial growth factor-A (VEGF-A), the sprouting process is directed by specialized ECs known as tip cells (Fig. 1), which are characterized by having long, dynamic filopodia [3]. With filopodia protrusions studded with VEGFR2 and other receptors, tip cells can sense their surroundings for guidance, which enables directional migration into avascular areas [3]. The ECs that follow the tip cells are known as stalk cells. Compared with tip cells, they produce fewer filopodia and instead proliferate to supply building blocks for growing sprouts [4]. Moreover, stalk ECs also create a vascular lumen, establishing tight junctions and adherens junctions to ensure the integrity of new sprouts, as well as depositing a basement membrane [5, 6]. Endothelial tip and stalk cells also display different gene expression patterns, with tip cells strongly expressing *Dll4*, *Vegfr2*, *Vegfr3*, *Pdgfb*, *Apelin, Cxcr4, CD34, Efnb2*, *Ang2*, insulin-like growth factor 2 (*Igf2*) and IGF-1-receptor (*Igf1r*) [7–11], with *Jag1*, *Robo4*, *Vegfr1* preferentially being expressed in stalk cells [12–14]. Once a branch vessel is formed, ECs become quiescent and are called “phalanx cells”, as they are aligned in a phalanx formation. They differ from both tip and stalk cells. Phalanx cells extend a few filopodias, migrate, deposit a basement membrane and establish junctions, similar to the characteristics of stalk cells [15–17]. However, they maintain quiescence and have limited proliferative capacity.

Due to sprouting at the vascular front being at the forefront of various physiological processes, understanding angiogenic sprouting is of primary interest for regulating both physiological and pathological angiogenesis. Moreover, the number and characteristics of tip cells determine the morphology and function of the finally-formed blood vessels [18]. Because of this significant role, the tip cells are a potential therapeutic target for disease treatment, either through therapeutic angiogenesis or anti-angiogenic therapies, which are needed for diseases such as cancer and various major eye diseases [19, 20]. Hence, research on the mechanisms of tip cell specification is necessary and of clinical significance. Closer examination of recent studies indicated that tip cell selection is a dynamic process of sprouting angiogenesis [21]. Therefore, it is necessary to integrate current information to further our understanding of tip cell specification. This review will concentrate on uncovering the underlying mechanisms of dynamic tip cell selection, particularly the role of cell rearrangement, tip cell selection signaling pathways and intercellular interactions.

## EC rearrangement in the dynamic process of tip cell selection

**Fig. 2:**
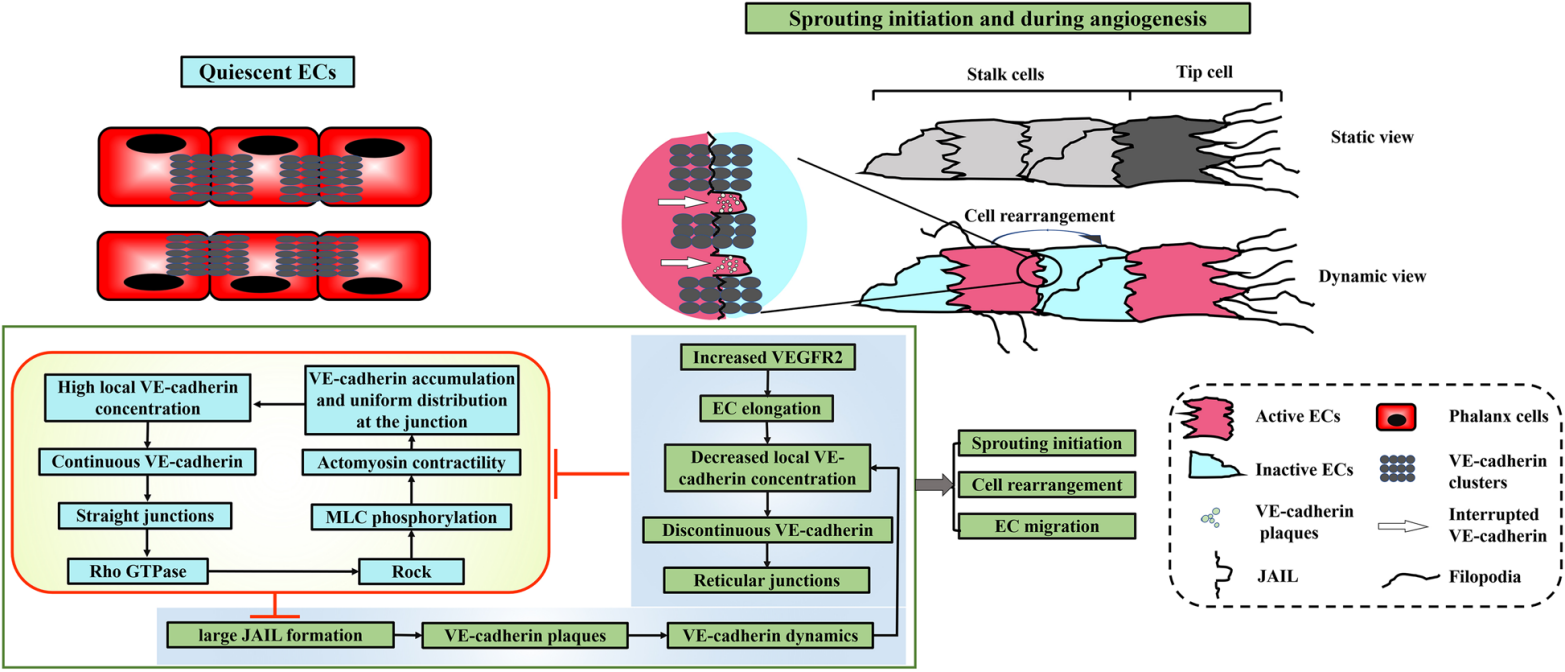
Different VE-cadherin and junctional patterns between quiescent and active ECs. In quiescent ECs, VE-cadherin is continuous and the cell-cell junction is straight, which leads to induction of MLC2 phosphorylation via Rho GTPase-ROCK activation. Actomyosin contractility is thus increased and eventually contributes to the uniform distribution of VE-cadherin at cell junctions. During angiogenesis, in the active cells (with higher VEGFR2 expression), VEGF induces elongation of ECs and thus results in decreased VE-cadherin concentration. Therefore, VE-cadherin is discontinuous and the cell-cell junction is reticular, which results in the disruption of the loop in quiescent ECs, as well as impairment of Junction associated intermittent lamellipodia (JAIL) formation. VE-cadherin dynamics, maintained by JAIL, are crucial for EC rearrangement and EC mobility

Tip cell specification involves a phenomenon termed EC rearrangement, which means that ECs within sprouting blood vessels dynamically shuffle and interchange their positions (Fig. 2) [13]. The discovery of EC rearrangement has challenged the traditional static view of the angiogenic process. It was previously thought that leading tip cells, once selected, would stay fixed at the head of the sprouts, leading the way for the following stalk cells to form the vascular lumen behind. On the contrary, due to EC rearrangement, the cellular state in a sprout is not unchangeable and various subtypes of ECs can overtake each other to compete for the tip sprout position during angiogenesis, thereby ensuring that the most competitive EC leads the sprout [13]. Hence in some way, tip cell competition rather than tip cell specification may more accurately reflect this dynamic process. Mechanistically, tip cell selection/competition requires both junctional dynamics and intercellular heterogeneity.

### Junctional VE-cadherin dynamics promote EC motility

In quiescent ECs, the cell-cell junction is straight, showing a continuous VE-cadherin expression pattern (Fig. 2). Normally, junctional VE-cadherin promotes MLC2 phosphorylation via ROCK activation by Rho-GTPase, most likely involving RhoC. As a result, actomyosin contractility is increased, leading to VE-cadherin being distributed uniformly at cell junctions in the established quiescent state [22]. Moreover, it had been proposed that the aforementioned feedback loop is negative for tip cell formation and angiogenesis by suppressing VEGFR2-dependent sprouting [22]. Therefore, sprouting initiation requires disruption of this negative feedback loop. Indeed, a moderate decline in VE-cadherin expression promotes angiogenic sprouting in vitro and within murine retina in vivo [22, 23].

However, it is still largely unknown how this negative feedback loop is interrupted at the beginning of sprouting and how the functional junction dynamics are maintained in the physiological process of angiogenesis. VE-cadherin-induced adherens junction dynamics and the consequent Junction Associated Intermittent Lamellipodia (JAIL) are implicated in this process. Specifically, higher VEGFR2 expression occurs only within a specific sub-population of sprouting ECs, displaying a salt-and-pepper pattern [13]. As shown in Fig. 2, when stimulated by a combination of VEGF and VEGFR2, ECs are elongated and this leads to a decline in the local VE-cadherin concentration. Therefore, in stark contrast to the straight and continuous junction, elongated ECs instead exhibit a reticular junction due to a disrupted VE-cadherin expression pattern [24, 25]. Consequently, the aforementioned feedback loop is interrupted and JAILs are formed at sites where local expression of VE-cadherin is lacking, via the involvement of the WAVE/WASP/ARP2/3 complex [24–26]. Formation of JAILs involves the appearance of lamellipodia-like structures at established endothelial junctions and are so named due to their spatio-temporal appearance [25]. Actin-driven JAIL overlaps with the corresponding plasma membrane of neighboring cells, at which VE-cadherin trans-adhesions are formed, and which appear as VE-cadherin plaques. These plaques are increasingly clustered during JAIL retraction and eventually incorporate VE-cadherin into the cell contacts. In this way, JAIL formation contributes to junctional VE-cadherin dynamics. This process is repeated until VE-cadherin concentration is sufficiently increased and JAIL formation is blocked.

With regards to VE-cadherin, it is important to note the following two points. Firstly, the regulation of junctional dynamics occurs at the subcellular level, rather than as a generalized behavior of the entire junctions. Disrupted VE-cadherin pattern and large JAIL formation are always observed at the cell poles, while continuous VE-cadherin expression patterns and occasional formation of small JAIL are characteristics of the lateral junctions. Secondly, it seems that cell elongation and decreased VE-cadherin expression are interdependent, forming a positive feedback loop [23]. Moreover, studies have demonstrated that the total expression of VE-cadherin remains stable within EC cultures in vitro and mice retinas, veins or the perivenous capillaries in vivo [24, 25]. Therefore, the initiation of sprouting and cell migration might be more complex than the regulation of local VE-cadherin concentration. Besides cell elongation, phosphorylation of VE-cadherin by VEGF, actin contractility, or other mechanisms on tyrosine residues leads to rapid and reversible endocytosis of VE-cadherin, thus weakening the adhesion level and promoting VE-cadherin turnover and mobility [22, 27]. In this way, phosphorylation of VE-cadherin might also contribute to the dynamic state of adherens junctions in ECs.

VE-cadherin dynamics and JAIL formation have two important functional effects: maintenance of junction integrity and promoting cell mobility. Cell junctions of large cells display large VE-cadherin-free spaces (white arrow in Fig. 2) between the individual VE-cadherin clusters and JAIL preferentially developed at these VE-cadherin-free spaces. Because JAIL overlapped plasma membranes of adjacent cells and allow formation of new VE-cadherin adhesion sites, this maintained monolayer integrity and controlled endothelial barrier function and remodeling [25, 26]. Additionally, owing to spatially restricted JAIL accumulation, VE-cadherin plaques are formed extensively at the leading end and anchor polarized stress fibers by adhesion, which contraction might guide the oriented migration of ECs [24]. The VE-cadherin dynamics-induced JAIL formation and stress fibers thus enable active cells to achieve mobility.

### Intercellular heterogeneity enables mobile ECs to interchange their positions

Cell rearrangement is based on interactions at a multi-cellular level, requiring some cells to move forward while others are left behind. That is, junctional dynamics and JAIL enable ECs to acquire mobility. Cell rearrangement only occurs when cell A (relatively inactive, the overtook cell), with lower VEGFR2 expression and junctional dynamics, is overtaken by the cell behind it (cell B, the overtaking cell), which is more active due to higher VEGFR2 expression and junctional dynamics. Recent studies have also demonstrated that the mode of rearranging cell motility also depends on differential adhesion between ECs [21, 28]. With increased heterogeneity between cell A and cell B, the possibility of cells overtaking each other would be improved. In the extreme case, cell B is also activated, and these two cells will lose heterogeneity and halt positional interchanges, in spite of their high activity levels. Hence, combining VE-cadherin dynamics and heterogeneity of different cells lays a foundation for angiogenic sprouting and cell rearrangement during angiogenesis.

As previously mentioned, tip cell competition is based on dynamic EC rearrangements. Mechanistically, ECs exhibit a high-level of heterogeneity with respect to the level of VEGFR2 expression [21]. The active cell sub-type (with higher VEGFR2 expression) achieves higher migration velocity towards the sprouting tip position due to cell elongation and JAIL formation, while those inactive ECs (with lower levels of VEGFR2) are less amenable to become tip cells [24, 29]. Altogether, cooperative interactions, functional junctional dynamics and intercellular heterogeneity would select the most competitive ECs to become the tip cell that leads the way for angiogenic sprouting.

## Role of signaling pathways and their interactive cross-talk in dynamic tip cell selection

**Fig. 3:**
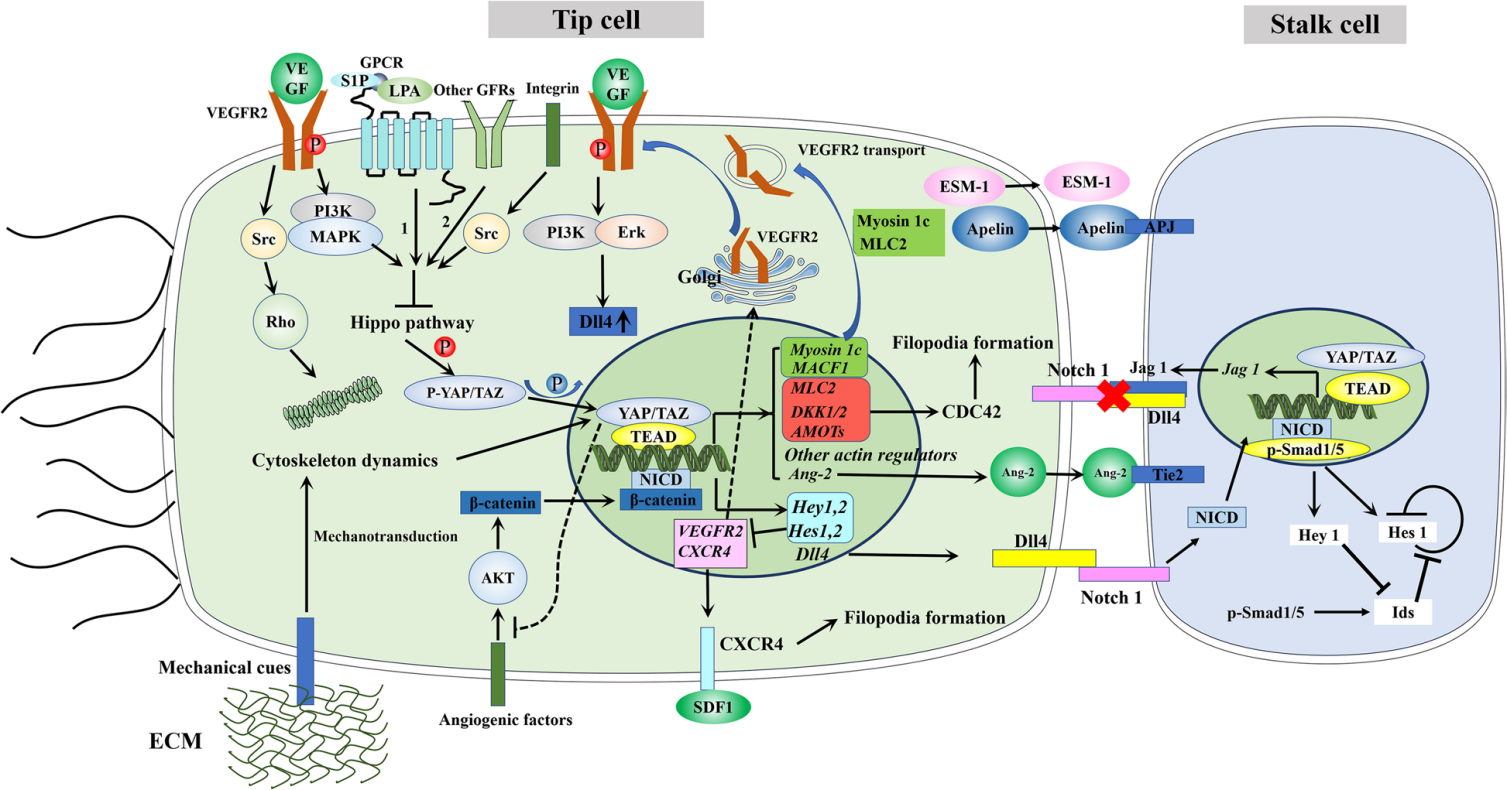
Overview of Notch Pathways, YAP/TAZ and their interaction in tip cell specification/competition. Notch signaling controls tip cell specification through ‘lateral inhibition’ [38]. Moreover, Notch signaling induces stalk cell phenotype through crosstalk with Smad1/5 [39]. Activation of VEGFR2, G-protein-coupled receptors (GPCRs), integrins and growth factor receptors (GFRs) results in YAP/TAZ activation through Hippo inhibition [40–42]. Moreover, cytoskeleton dynamics induced by VEGFR2-Src-Rho and ECM mechanical cues also drives YAP/TAZ activation [43, 44]. YAP/TAZ promotes filopodia formation by sustaining activation of the Rho family GTPase Cdc42 by increasing the expression of downstream target genes, including *DKK-1/2*, *Mlc2*, and *AMOTs* [45]. Moreover, some secreted molecules expressed by tip-enriched genes, including *Ang-2, Apelin* and *endothelial-specific molecule 1*(*ESM-1*), were produced by tip cells and guide the behavior of following stalk cells in a paracrine manner [9]. Notch signaling intertwines with YAP/TAZ through VEGF signaling. VEGF signaling activates YAP/TAZ and Notch signaling [44]. In turn, YAP/TAZ-dependent expression of several cytoskeletal remodeling genes, including *myosin 1c* and *MACF1*, are implicated in trafficking VEGFR2 from the Golgi apparatus to the plasma membrane [46]. Notch signaling suppression in tip cell leads to increased VEGFR2 expression [47]. (The red and blue letter P indicates phosphorylation and dephosphorylation, respectively. Dotted lines indicate no crossing with the solid lines below them. 1 and 2 refre to Lats inhibition and Ras-Raf-MAPK, respectively)

Tip cell selection involves both cell-cell and cell-extracellular matrix (ECM) interactions. With regards to cell-cell interactions, the list of known signaling molecules capable of regulating tip-stalk specification is steadily growing, the ‘‘principal player’’ being the Notch signaling pathway, of which inhibition has been shown to dramatically enhance tip cell formation and sprouting angiogenesis. Previous studies have reported that microRNA-30 (miR-30) [30], activin receptor-like kinase 1 (ALK1) [31], transcription factor NF-E2–related factor 2 (Nrf2) [32] and SRY-related HMG box 17 (SOX17) [33] have been identified as key regulators of tip cell selection, but they all act directly on either the Notch signaling pathway itself, or downstream of it. By contrast, the interaction between the ubiquitous ECM environment and ECs is underappreciated and understudied and has not been given sufficient attention in research on tip cell specification. To date, only endothelial basement membrane components [34], or cytokines such as Tumor Necrosis Factor (TNF) [35], VEGF [36], and Yes-associated protein (YAP)/ transcription activator with PDZ binding motif (TAZ) [37], which are all sensors of the microenvironment, have been reported to mediate tip cell/stalk cell specification. Mechanistically, the basement membrane, TNF and VEGF, similarly control tip cell formation through modulation or cross-talk with the Notch signaling pathway, while YAP/TAZ might exert their effects through actin cytoskeleton remodeling. Therefore, in this section, we will mainly focus on the effects of the Notch and YAP/TAZ signaling pathways as well as their interaction on tip cell selection, which are summarized in Fig. 3.

### The role of the notch signaling pathway in regulating tip cell number and morphology

**Fig. 4:**
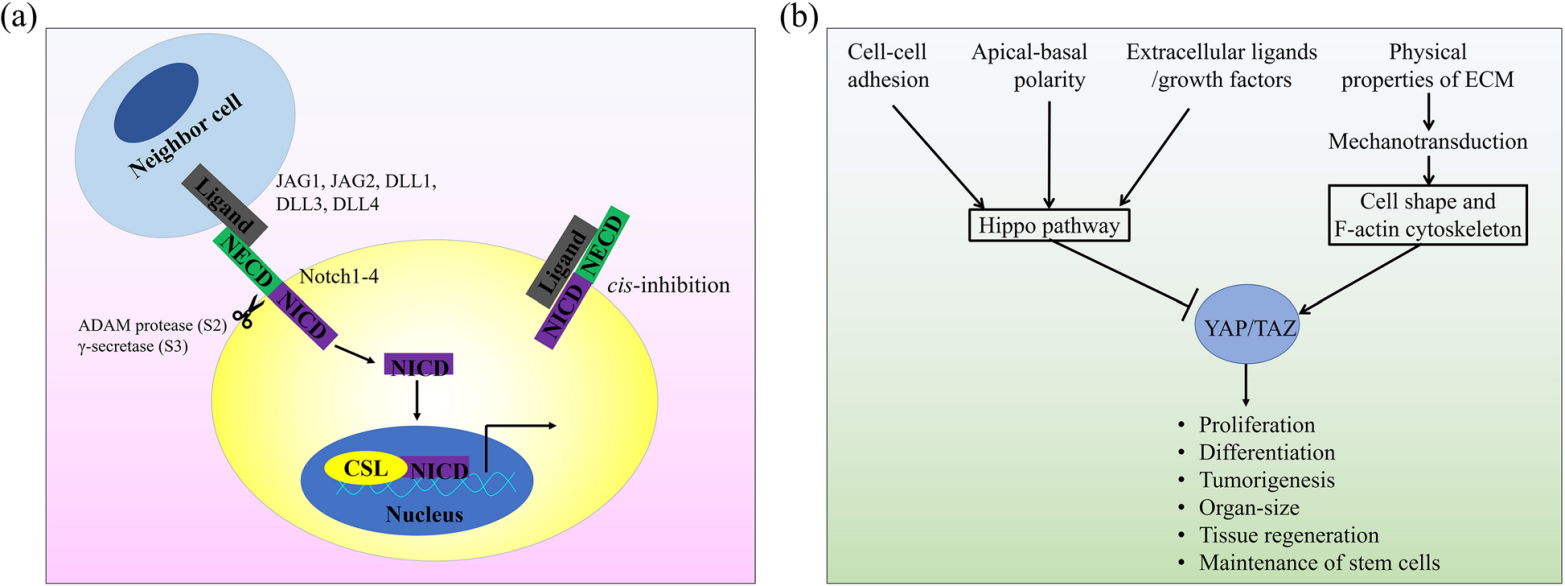
Overview of Notch and YAP/TAZ signaling pathways (**a**) Notch signaling cascade. Notch pathway is activated by ligands expressed on the neighboring cell, a process called trans-interaction. Alternatively, when Notch ligands and receptors are co-expressed in the same cell, they will interact, resulting in cis-inhibition. **b** Schematic representation of YAP and TAZ (YAP/TAZ) regulatory inputs and biological functions. YAP/TAZ can be activated both by Hippo pathway inhibition and in a Hippo-independent manner. Activated YAP/TAZ are translocated into the nucleus, where they regulate genetic programs

The Notch pathway is evolutionarily conserved and implicated in diverse biological functions, such as cell differentiation, survival, stem cell behavior and normal embryonic development [48]. Activation of the Notch signaling pathway is initiated by the interaction between the Delta–Serrate–Lag (DSL) and Notch protein receptors, which are expressed on the membranes of two neighboring cells, respectively (Fig. 4a). In mammalian ECs, there are four canonical DSL (Delta, Serrate, LAG-2) ligands: Delta-like 1 (Dll1), Delta-like 4 (Dll4) Jagged-1 (Jag1), and Jagged-2 (Jag2) and two Notch receptors: Notch1 and Notch4. Binding of a DSL ligand to the extracellular domains of the Notch receptor (NECD) triggers the canonical pathway of *in trans* Notch signaling. Specifically, binding initiates a series of proteolytic cleavages of the Notch receptor, first within the juxtamembrane region by a member of the disintegrin and metalloproteases family, followed by γ-secretase within the transmembrane domain, leading to release of the Notch intracellular domain (NICD) from the cell membrane. Then, NICD translocates to the cell nuclei, where it directly binds the transcription factor CSL, which turns on expression of Notch target genes such as *the basic helix-loop-helix (bHLH) proteins Hairy/Enhancer of Split (Hes)*, *Notch-regulated ankyrin repeat protein (Nrarp)*, and *Hes-related proteins (Hey/HRT/HERP)* [49–52].

#### Notch signaling pathway and tip cell numbers

Notch signaling controls tip cell specification through ‘lateral inhibition’, by which small differences in Notch activity between adjacent cells are amplified. Mechanistically, after combination with VEGFR2, VEGF-A stimulates ECs in hypoxic regions to express the Notch ligand Dll4 through phosphoinositide 3 kinase (PI3K) and Erk signaling [32, 53]. Dll4 then activates Notch signaling in adjacent cells, as well as NICD promoters downstream of target gene expression, which encode proteins that act as transcriptional repressors of, for example, *VEGFR2/3*, *Dll1*, *Dll4*, and *Jag1* [47, 54]. Therefore, neighboring cells exhibit reduced sensitivity to VEGF stimulation due to downregulation of the VEGF receptor and exert less lateral inhibition to its adjacent cell due to reduced expression of Dll4. As a result, the cell with higher Dll4 and lower Notch activity are more competitive and become tip cells, while the neighboring cell with lower Dll4 and higher Notch activity becomes stalk cells [55]. Several studies have reported that the Notch pathway activated by Dll4 may promote stalk cell specification and negatively regulates endothelial tip cell formation and vessel branching [38, 56]. Inhibition of Notch pathway with γ-secretase inhibitor or Dll4 blocking will markedly increase tip cell number and phenotype, resulting in a hyperbranched vessel. Mechanistically, the Notch pathway promotes stalk cell phenotype and specification through interactive crosstalk with Smad1/5 [39]. In ECs, activated Smad1 and Smad5 directly potentiate downstream target gene expression of the Notch signaling pathway by forming a complex with NICD [57]. Moreover, members of the Id family (inhibitors of cell differentiation or inhibitors of DNA-binding, encoding members of the helix-loop-helix (HLH) family of transcription factors), downstream of Smad signaling, are known to suppress the DNA-binding activity of Hes1 by the formation of heteromers with Hes1 at their HLH domain, thereby releasing the negative autoregulatory loop of Hes1 from its own promoter and augmenting Hes1 levels in the endothelium [58]. This in turn increased Notch signaling levels by Smad1/5 and decreased Id protein levels through Hey1-mediated Id degradation, thus attenuating the Notch signaling in stalk cells and rendering them non-responsive to cell shuffling and preventing them from acquiring tip cell characteristics [57]. Specific inactivation of Smad1/Smad5 in the ECs of mouse embryos results in impaired Notch signaling and promotes increasing numbers of tip-cell-like cells, indicating a model where crosstalk between Notch and Smad1/5 orchestrates tip cell/stalk cell selection [39]. Taken together, the integration of Notch and Smad1/5 signaling cascades modulates stalk cell phenotype and tip cell inhibition.

#### Notch signaling pathway and tip cell morphology

In sprouting angiogenesis, tip cells are characterized by numerous filopodia, which are involved in a number of cellular processes such as guidance towards chemoattractants, adhesion to extracellular matrices, and cell migration. Hence, filopodia is necessarily required for tip cells to function normally. Studies have found that suppression of Notch signaling by γ-secretase inhibitor (GSI) treatment or genetic deletion of one Dll4 allele [38] markedly enhances the number and length of tip cell filopodia, whereas overactivation of Notch signaling by overexpression of the NICD [59] and full-length Dll4 [60] reduces the filopodia formation and migratory behavior of ECs. Hence, Notch signaling influences both the quantity and the quality of tip cells. The role of Notch inhibition in promoting filopodia formation can be explained by the downregulation of VEGFR2 and other receptors and consequent impaired EC motility by making them less responsive to VEGF [14]. Additionally, suppression of the Dll4-Notch pathway leads to upregulation of the chemokine receptor CXCR4 [61], which strongly promotes filopodia protrusion of tip cells upon interaction with its ligand stromal-cell derived factor-1 (SDF-1) [10]. Although both the VEGF-VEGFRs and SDF-1/CXCR4 signaling axis might partly account for the underlying mechanisms by which Notch signaling regulates filopodia formation and EC motility, more investigations are needed in order to deepen our understanding of this process.

### The role of YAP/TAZ signaling in regulating tip cell number and morphology

Since the discovery of YAP about two decades ago, the YAP/TAZ signaling pathway (Fig. 4b) has attracted much attention from scientists and researchers, with studies on the physiological functions and regulatory mechanisms of YAP/TAZ having become a major field in biological science research. Genetically, YAP and TAZ have been linked to a ubiquitous system (Hippo signaling pathway) that control the growth of organs until they reach their correct size [62]. Moreover, YAP/TAZ have been implicated in many signal transduction pathways that regulate metabolism, development, positional sensing, tissue regeneration and tumorigenesis [63]. YAP/TAZ are mainly understood to be downstream effectors of the Hippo pathway, a kinase cascade which ends in the phosphorylation and inhibition of YAP/TAZ, causing their cytoplasmic sequestration, degradation, and inactivation [64, 65]. Multiple signaling pathways and multiple extracellular ligands/growth factors have been implicated in regulating the Hippo pathway, including ligands of G-protein-coupled receptors (GPCRs) through inhibit the Hippo pathway kinases Lats1/2 [41], integrins [41], and Epidermal growth factor (EGF) through Ras-Raf-Mitogen activated protein kinases (MAPK) signaling axis induced by the EGF-receptor (EGFR) [40]. However, activation of YAP/TAZ in a Hippo-independent manner, mainly relies on mechanotransduction transmitted by the cytoskeletal system, which has also been reported to attract much interests [43, 66, 67]. YAP and TAZ are transcriptional coactivators that upon activation, would translocate from the cytosol to the nucleus where they interact with DNA-binding transcription factors, mainly the TEA-domain family member (TEAD 1–4) [68, 69]. In this manner, the YAP/TAZ-TEAD protein complex regulates the expression of multiple genes that control cell differentiation, proliferation and apoptosis.

#### YAP/TAZ signaling and tip cell numbers

Cell-cell adhesion and apical-basal polarity have also been proposed as regulators of the Hippo signaling cascade that determine YAP/TAZ localization and phosphorylation. In stable, quiescent blood vessels, phalanx cells typically form a cobblestone-like monolayer and line the luminal surface of the vasculature, with cell-cell junctions being effectively provided by adhesive interactions at adherens and tight junctions [21, 70]. YAP/TAZ in quiescent phalanx cells is inhibited by vascular endothelial cadherin-induced Hippo pathway activation [71]. Once stimulated by VEGF, vascular VE-cadherin of phalanx cells are phosphorylated, which subsequently triggers disruption of its assembly and induces instability of the AJ protein [45]. Similarly, angiogenic stimuli also induces ablation of TJ proteins. This results in disruption of cell-cell adhesions in ECs, leading to inhibition of the Hippo signaling pathway, which reduces the phosphorylation of YAP/TAZ and increases its nuclear localization, hallmarks of YAP/TAZ activation. Moreover, VEGF can activate YAP/TAZ through different intermediary mechanisms, including the VEGF-VEGFR2-Src-RhoGTPase signaling axis, leading to inhibition of signal transducer and activator of transcription 3 (STAT3) and Hippo signaling via VEGF-VEGFR-mediated phosphatidylinositol 3-kinase (PI3K)/mitogen-activated protein kinase (MAPK) activation [72–74]. In return, the activated YAP/TAZ further enhances the structural disorganization of AJs and TJs by controlling the expression of appropriate gene subsets, which thus forms a feed-forward loop. Since the loosening of EC junctions is the first step of angiogenesis, followed by initiation and expansion of angiogenic sprouting, YAP/TAZ might play a major role in the selection of EC candidates for tip cells. Additionally, as sensors of the microenvironment, YAP/TAZ translates mechanical stimuli into biochemical signals through mechanotransduction. Hence, mechanical cues, such as stiffness in pathological conditions, are implicated in angiogenesis regulation via YAP/TAZ mediated mechanotransduction.

#### YAP/TAZ signaling and tip cell morphology

Similar to the Notch signaling pathway, YAP/TAZ signaling also plays an important role in regulating filopodia formation of tip cells. One study [37] observed a blunted-end, aneurysm-like tip ECs with fewer filopodia in *Yap/Taz* ^*iΔEC*^ mice. Upon hyperactivation of YAP/TAZ, excessive filopodia protrusions could be observed in tip ECs. Moreover, the tip cells in Yap/Taz deleted mice had no organized F-actin bundle–containing protrusions, indicating that YAP/TAZ play crucial roles in filopodia formation by regulating the rearrangement of cytoskeletal proteins. Mechanistically, it is thought that YAP/TAZ modulates the actomyosin cytoskeleton by sustaining activation of the Rho family GTPase Cdc42, which in turn upregulates the expression of downstream target genes, including *DKK-1/2*, *Mlc2*, and *AMOTs* [37, 45]. Cdc42 is critically required for filopodia formation in the ECs of angiogenic sprouts [75, 76], and studies have shown that deletion of Cdc42 caused severe defects in endothelial migration [77]. Moreover, YAP/TAZ activation might also enhance tip cell phenotype by promoting the expression of MLC2 [37], a component of non-muscle myosin II that plays key roles in cell adhesion, migration, and tissue morphogenesis [78].

Recent studies have revealed that nuclear localization of YAP/TAZ was observed in vascular front ECs during angiogenesis [73, 79] and that tip/stalk specification is connected with the on/off system of YAP/TAZ signaling. Deletion of YAP/TAZ in mice has resulted in obvious defects in the qualities of tip cells, spouts and branching, while hyperactivation of YAP/TAZ leads to excessive branches and hyperplastic vascular growth in *Lats1/2* ^*iΔEC*^ mice [37]. However, although the involvement of YAP/TAZ activation in tip cell specification is indisputable, investigations on the underlying regulatory mechanisms are limited. Nevertheless, it can be proposed that YAP/TAZ activates EC to acquire a tip cell phenotype. Angiopoietin 2 (Ang-2), being transcriptionally regulated by YAP [73, 75], is found to be a tip-enriched gene [9, 10], with the corresponding protein being specifically expressed at the tips of invading EC sprouts [80], thus confirming the role of Ang-2 in promoting tip cell formation. This logical deduction was further validated by the study of Winnik et al. [81], which found that Ang-2 overexpressing ECs exhibited more sprouting when compared with the control ECs. Mechanistically, it is suggested that Ang-2 converts blood vessels into a more plastic and immature phenotype by blocking constitutive tie-2 activation induced by Ang-1, thus further enhancing sprouting required for neovascularization initiation [81].

It is notable that besides direct cell-cell interaction, the tip-stalk can also cross-talk through paracrine signaling. Similar to *Ang-2*, some secreted molecules expressed by tip-enriched genes, including *Apelin* and *endothelial-specific molecule 1*(*ESM-1*) are produced by tip cells and secreted into the ECM [9]. Since receptors of these proteins are preferentially expressed on the stalk cells, the tip cell may guide behavior of following stalk cells in a paracrine manner. Considering that tip cell selection is a competitive dynamic process, this indirect interaction between tip cells and stalk cells, might presumably also contribute to dynamic competition during angiogenesis. However, the above hypothesis needs to be further validated by future studies.

### Notch and YAP/TAZ signaling pathways are intertwined in the process of tip cell selection

Notch signaling has been reported to crosstalk with the Hippo signaling pathway and modulate YAP/TAZ activity in different cell types [82]. However, limited studies have been performed on ECs and further investigations are thus required to determine whether each independent stimulus results in a specific, or overlapping, YAP/TAZ-dependent transcriptional output, and whether this also varies between different EC types. Based on current studies, we propose that Notch and YAP/TAZ signaling might interact by the VEGF pathway and there might be YAP/TAZ-dependent transcriptional regulation of Notch ligands.

#### The possible interactive cross-talk between the Notch, YAP/TAZ and VEGF signaling pathways

Recent studies have used different approaches to confirm that Notch and YAP/TAZ signaling are key effectors of VEGF-mediated angiogenesis [14, 20, 37, 79]. In fact, both Notch and YAP/TAZ signaling crosstalk with the VEGF signaling pathway can regulate the angiogenic capacity of ECs and modulate their levels of signaling. During angiogenesis, VEGF activates Notch signaling and YAP/TAZ through upregulation of the ligand Dll4, as well as through VEGF-induced actin cytoskeleton changes, respectively. It is worth pointing out that increased activity of the Notch signaling pathway occurs in neighboring cells adjacent to those stimulated by VEGF, with YAP/TAZ signaling in the same cell. In turn, the activated Notch signaling pathway downregulates the expression of VEGFR2, leading to decreased sensitivity of ECs to VEGF. Ultimately, through lateral inhibition of Notch signaling, the cell that receives the strongest stimulation by VEGF expresses the highest level of VEGFR2. Similar but not exactly the same, hyperactivated YAP/TAZ signaling by VEGF can also promote VEGF-VEGFR2 signaling by regulation of VEGFR2 subcellular localization rather than through transcriptional output. Specifically, YAP/TAZ-dependent expression of several cytoskeletal remodeling genes, including *myosin 1c* and *MACF1*, are implicated in trafficking VEGFR2 from the Golgi apparatus to the plasma membrane [46, 79]. Accordingly, although it still remains to be validated, we hereby speculate that Notch and YAP/TAZ signaling are intertwined through the VEGF signaling pathway.

#### YAP/TAZ inhibits Notch signaling by modulating the expression of Notch ligands

In general, there are two main models by which direct YAP/TAZ-Notch signaling interaction have been reported: YAP/TAZ-dependent transcriptional regulation of Notch ligands or receptors; and physical interaction of YAP/TAZ with NICD to regulate transcription of common target genes [82]. However, the joint transcriptional co-regulation by YAP/TAZ and NICD have also been proposed in other cell types, such as vascular smooth muscle cells, other than ECs [83]. Therefore, we will mainly focus on the regulation of the Notch signaling pathway by YAP/TAZ.

Nuclear YAP/TAZ leads to suppression of Notch signaling in ECs through downregulation of Dll4. Consistent with these findings, Neto et al. [84] also found that knockdown of YAP/TAZ resulted in increased Dll4 expression, which in turn led to substantial enhancement of Notch activity and corresponding target gene expression. Mechanistically, it can be excluded that decreased Dll4 is the direct cause of YAP/TAZ activation because nuclear YAP/TAZ suppresses Dll4 in a TEAD–independent manner. Yasuda et al. [85] proposed a molecular mechanism involving active YAP/TAZ repressing NICD- and β-catenin-mediated Dll4 induction by inhibiting the Akt signaling pathway. This proposal is plausible given that β-catenin and NICD are indispensable for Akt-induced Dll4 expression in ECs [86, 87]. Additionally, recent studies have suggested that Dll4 and Jag1 have opposite effects during sprouting angiogenesis [12, 88, 89]. Contrary to Dll4, Jag1 acts as a pro-angiogenic regulator of sprouting and dramatically enhances tip cell formation because of its ability to antagonize the Dll4-Notch pathway [12]. Indeed, the loss of Jag1 in ECs leads to a marked decline in the number of tips and filopodia, while Jag1 overexpression resulted in the opposite effect [54, 88, 90]. Interestingly, Jag1 is one of the YAP/TAZ/TEAD-dependent transcriptional genes [91]. Accordingly, although it still remains to be investigated, we can make an informed conjecture that activated YAP/TAZ might promote tip cell specification, also through Jag1-mediated Notch signaling inhibition.

## Cooperation between EC rearrangement and tip cell selection signaling pathways during the dynamic process of tip cell selection

**Fig. 5:**
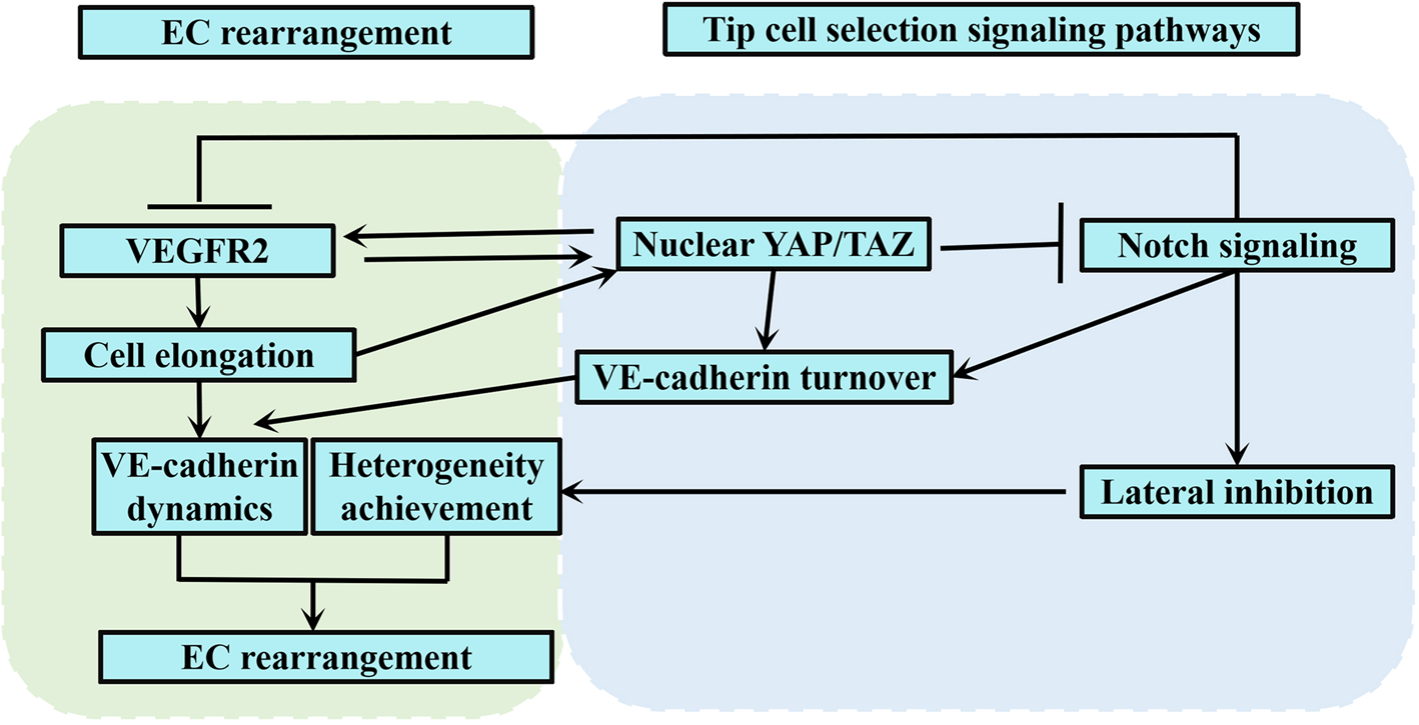
Interaction of EC rearrangement and tip cell selection signaling pathways

EC rearrangement and tip cell selection signaling pathways are closely intertwined (Fig. 5). Notch signaling pathway enables neighboring cells to achieve heterogeneity through lateral inhibition and is thus required in angiogenesis to drive the normal rearrangement of ECs [21, 28]. Moreover, nuclear localization of YAP/TAZ facilitates VE-cadherin induced junctional dynamics through promotion of VE-cadherin turnover activity and upregulation of transcriptional expression and membrane distribution of VEGFR2 [79, 84]. In turn, cell elongation and formation of stress fibres, resulting from higher VEGFR2 expression and decreased VE-cadherin concentration, might facilitate translocation of YAP/TAZ from the cytoplasm to the nucleus, thus activating the YAP/TAZ signaling pathway [24, 92]. Altogether, since membrane levels of VEGFR2 are increased in ECs by extracellular VEGF through Notch signaling and nuclear YAP/TAZ translocation, EC rearrangement combines VEGF stimulation with EC competitiveness to ensure the cell closest to the highest VEGF concentration gains the most competitive advantage to become the leading cell of a vascular spout, thus improving the efficiency of angiogenesis.

Besides selecting the most competitive EC in a sprout to be the leading cell, it seems that the combination of EC rearrangement with tip cell selection signaling pathways might also contribute to balancing the tip cell number. It has been demonstrated that active ECs during angiogenic sprouting can either form a new sprout or shuffle up through the existing branch to compete for the tip position [21]. Moreover, it has also been shown that when activated ECs in a sprout are interconnected, they will lose heterogeneity and stop changing their position [21]. Consequently, although the regulatory factors of such a branch-or-shuffle decision process amongst the activated cells are unknown, it can be speculated based on the mechanism of angiogenesis and cell rearrangement, that when interconnected active ECs halt interchange positions for a prolonged period, they might attune to the surrounding tissues as a new branch due to their mobility, leading to increased tip cell numbers. Moreover, since factors that upregulate junctional dynamics will improve the number of active ECs, it might be possible, to some extent, that when two or more active cells are connected, heterogeneity is lost together with the ability to interchange positions during the process of angiogenesis. Consistently, using computational modelling and experimental mouse pathology models, it has been documented that under high VEGF conditions, contiguous regions of all-active or all-inhibited states are larger, resulting in halting of positional interchanges and rearrangement defects [28, 93]. Accordingly, it is understandable that any factors promoting VE-cadherin dynamics or loss of heterogeneity, including upregulating VEGFR2 level in the membrane, increasing VEGF concentration, knocking down or blocking of VE-cadherin, Notch signaling inhibition, and YAP/TAZ nuclear localization etc., contributes to enhancement of tip cell formation and hypersprouting, which is consistent with the phenomenon observed in previous studies [23, 28].

Taken together, through Notch signaling-induced heterogeneity, mechanotransduction by YAP/TAZ signaling, VEGFR2-mediated tip cell competition, cooperation of EC rearrangement and tip cell selection signaling pathways, various mechanical and chemical signals are integrated to secure the right balance between tip and stalk cells and establish the right number of cells at the right place during angiogenesis, thus orchestrating the morphogenic behaviors that ensure correct vessel patterning.

## Future perspectives

In this review, we focus on the roles of EC rearrangement and tip cell selection signaling pathways, in particular the Notch and YAP/TAZ signaling pathways, together with their cooperative interactions, as a comprehensive way to understand the mechanisms of tip cell selection/competition as a dynamic process. Nevertheless, despite rapid progress in recent years, several outstanding questions remain unanswered.

The aforementioned lateral inhibition is based on *in trans* Notch signaling, involving expression of the two ligands in separate cell populations (i.e., Dll4 in signal-sending and Notch1 in signal-receiving cells). However, contrary to non cell-autonomous lateral inhibition, several studies have proposed that Notch ligands and receptors can autonomously inhibit signaling by being co-expressed in the same cell, termed *cis*-inhibition [94]. This Notch ligand-receptor interaction protects a cell from receiving lateral inhibition signaling from ligands expressed by adjacent cells, thus serving as a defense mechanism against cell-autonomous Notch receptor activation and reinforcing lateral inhibition, as a threshold-setting system [82, 95, 96]. Moreover, it has been shown that *cis*-inhibition is also implicated in determining epidermal stem cell fate [97]. Due to the significance of *cis*-inhibition and the remarkable parallels and similarities between the signaling regulatory mechanisms of ECs and epithelial cells, further studies are needed to determine whether Notch *cis*-inhibition occurs in ECs, and if so, how these exert roles during the tip cell selection and angiogenesis.

During embryonic development, the physical properties of ECM and mechanical forces are indispensable to morphogenetic processes, including tissue architecture definition and specific cell differentiation specification [98]. Mechanotransduction enables cells to sense biomechanical signals and transduce these into biochemical signals to adapt to the microenvironment [99]. Hence, it is promising to propose that physical cues from ECM and mechanotransduction might also account for tip cell formation during angiogenesis. Indeed, it has been documented recently that matrix stiffening leads to hyperbranching in vitro and in vivo [100]. The response of YAP/TAZ to different mechanical inputs has highlighted its key role as universal mechanotransducers and mechanoeffectors [92]. However, the mechanotransduction of YAP/TAZ in tip/stalk specification/competition, particularly extracellular mechanical signaling, has not yet been characterized extensively. Therefore, further studies are needed to deepen our understanding of the mechanisms of angiogenesis.

Finally, although Notch signaling inhibition increases tip cell numbers and eventually results in the formation of a denser and more highly interconnected superficial capillary network, the vessels formed are immature, resulting in tissue perfusion being attenuated [14, 19]. Therefore, it is a challenge to strike the right balance between the tip cell number and the formed functional and perfused vessels, especially in therapeutic angiogenesis. Hence, more precise spatiotemporal control of Notch activity is required during angiogenesis. It is possible that YAP/TAZ induced mechanotransduction mechanisms might offer an alternative, for example, via spatiotemporal adjustment of ECM stiffness. It is possible that combined manipulation of Notch signaling and YAP/TAZ induced mechanotransduction might achieve better therapeutic efficacy, which needs to be verified by future studies.

## Conclusions

This review focuses on critically examining the underlying mechanisms of dynamic tip cell selection, particularly the role of cell rearrangement, tip cell selection signaling pathways and intercellular interactions. Various mechanical and chemical signaling cues are integrated to ensure the right number of cells at the right place during angiogenesis, thereby precisely orchestrating morphogenic functions that ensure correct patterning of blood vessels.

## Data Availability

Not applicable.

## Abbreviations

ECs: Endothelial cells
VEGF-A: Vascular endothelial growth factor-A
Igf2: Insulin-like growth factor 2
Igf1r: IGF-1-receptor
JAIL: Junction Associated Intermittent Lamellipodia
ECM: Extracellular matrix
MiR-30: MicroRNA-30
ALK1: Activin receptor-like kinase 1
Nrf2: Transcription factor NF-E2–related factor 2
SOX17: SRY-related HMG box 17
TNF: Tumor Necrosis Factor
YAP/TAZ: Yes-associated protein / transcription activator with PDZ binding motif
DSL: Delta–Serrate–Lag
Dll1: Delta-like 1
Dll4: Delta-like 4
Jag1: Jagged-1
Jag2: Jagged-2
NICD: Notch intracellular domain
bHLH: basic helix-loop-helix
Hes: Hairy/Enhancer of Split
Nrarp: Notch-regulated ankyrin repeat protein
Hey/HRT/HERP: Hes-related proteins
PI3K: Phosphoinositide 3 kinase
GSI: γ-secretase inhibitor
SDF-1: Stromal-cell derived factor-1
GPCRs: G-protein-coupled receptors
EGF: Epidermal growth factor
EGF-R: Epidermal growth factor- receptor
TEAD 1–4: TEA-domain family member
STAT3: Signal transducer and activator of transcription 3
PI3K/MAPK: Phosphatidylinositol 3-kinase /mitogen-activated protein kinase
Ang-2: Angiopoietin 2
ESM-1: Endothelial-specific molecule 1
